# *Trypanosoma cruzi* pathogenicity involves virulence factor expression and upregulation of bioenergetic and biosynthetic pathways

**DOI:** 10.1080/21505594.2022.2132776

**Published:** 2022-10-25

**Authors:** Juan San Francisco, Constanza Astudillo, José Luis Vega, Alejandro Catalán, Bessy Gutiérrez, Jorge E Araya, Anibal Zailberger, Anabel Marina, Carlos García, Nuria Sanchez, Antonio Osuna, Susana Vilchez, Marcel I Ramírez, Janaina Macedo, Verónica Santiago Feijoli, Giuseppe Palmisano, Jorge González

**Affiliations:** a Molecular Parasitology Unit, Medical Technology Department, University of Antofagasta, Antofagasta, Chile; b Laboratory of Gap Junction Proteins and Parasitic Disease, Instituto Antofagasta, Universidad de Antofagasta, Antofagasta, Chile; c Research Center in Immunology and Biomedical Biotechnology of Antofagasta, Antofagasta, Chile; d National University of La Plata, La Plata, Argentina; e Centro de Biología Molecular Severo Ochoa Universidad Autonoma de Madrid, Madrid, Spain; f Institute of Biotechnology, University of Granada, Granada, Spain; g Laboratório de Biologia Molecular e Sistemática de Trypanosomatides, Instituto Carlos Chagas, Fiocruz, Parana, Brazil; h Department of Parasitology, University of Sao Paulo, Sao Paulo, Brazil; i Laboratório de Biologia Molecular e Sistemática de Trypanosomatides, Millennium Institute on Immunology and Immunotherapy, Antofagasta, Chile

**Keywords:** *Trypanosoma cruzi*, virulence, genetically related cell lines, proteomics

## Abstract

The molecular repertoire of *Trypanosoma cruzi* effects its virulence and impacts the clinical course of the resulting Chagas disease. This study aimed to determine the mechanism underlying the pathogenicity of *T. cruzi*. Two *T. cruzi* cell lines (C8C3***hvir*** and C8C3***lvir***), obtained from the clone H510 C8C3 and exhibiting different virulence phenotypes, were used to evaluate the parasite’s infectivity in mice. The organ parasite load was analysed by qPCR. The proteomes of both *T. cruzi* cell lines were compared using nLC-MS/MS. Cruzipain (Czp), complement regulatory protein (CRP), trans-sialidase (TS), Tc-85, and sialylated epitope expression levels were evaluated by immunoblotting. High-virulence C8C3***hvir*** was highly infectious in mice and demonstrated three to five times higher infectivity in mouse myocardial cells than low-virulence C8C3***lvir***. qPCR revealed higher parasite loads in organs of acute as well as chronically C8C3***hvir***-infected mice than in those of C8C3***lvir***-infected mice. Comparative quantitative proteomics revealed that 390 of 1547 identified proteins were differentially regulated in C8C3***hvir*** with respect to C8C3***lvir***. Amongst these, 174 proteins were upregulated in C8C3***hvir*** and 216 were downregulated in C8C3***lvir***. The upregulated proteins in C8C3***hvir*** were associated with the tricarboxylic acid cycle, ribosomal proteins, and redoxins. Higher levels of Czp, CRP, TS, Tc-85, and sialylated epitopes were expressed in C8C3***hvir*** than in C8C3***lvir***. Thus, *T. cruzi* virulence may be related to virulence factor expression as well as upregulation of bioenergetic and biosynthetic pathways proteins.

## Introduction

The protozoan parasite *Trypanosoma cruzi* is the causal agent of Chagas disease, a public health problem affecting 6–8 million people worldwide [1]. Of these, millions of patients with chronic infections are at a further risk of developing cardiac pathologies, thus rendering Chagas disease as one of the main causes of heart disease and early mortality, especially in South America [2].

*T. cruzi* virulence involves metacyclogenesis, which allows the transformation of the parasite into metacyclic forms that infect different cell types as well as proliferate and evade the host immune response [3]. Several *T. cruzi* proteins involved in virulence and immune evasion have been described, and are collectively denoted as virulence factors. Amongst these, cysteine proteases such as cruzipain (Czp), and members of the Gp85/trans-sialidase (TS) family, including Tc-85 (a surface glycoprotein specific to the trypomastigote stage of *T. cruzi*), complement regulatory protein (CRP), and TS, are well-known *T. cruzi* virulence factors [4,5]. Although the specific role of each virulence factor remains uncertain, experimental evidence has led to a comprehensive and integrated understanding of the impact of virulence factors on *T. cruzi* infection [5].

Elucidating the virulence of *T. cruzi* is challenging because of the parasite’s intricate biology, including unique gene expression mechanisms, an absence of processes regulating the initiation of transcription, and the existence of two distinct infective stages in the vertebrate host: the metacyclic trypomastigotes (MTs) stage and the bloodstream trypomastigotes stage [6]. Furthermore, *T. cruzi*, being a genetically heterogeneous parasite species, comprises a wide variety of strains and isolates vectored by different species of triatomines and therefore capable of infecting varied warm-blooded vertebrates [7]. This significant biological diversity is associated with differences in virulence and clinical symptoms across strains [8]. Currently, *T. cruzi* is classified into seven discrete typing units (DTUs), including TcI – VI and Tcbat [2,9]. Although pathogenicity may be modulated through diverse mechanisms in various *T. cruzi* strains [5], the higher virulence of some strains could be attributed most likely to the differential expression of some virulence factors [5]. Numerous parasitic components have been suggested to participate in this pathogenic mechanism, however, the molecular basis of the pathogen’s virulence remains unclear [3].

Several studies have compared high- and low-virulence *T. cruzi* strains as well as clones derived from the same *T. cruzi* strain [3,10–13], but no definitive evidence has been obtained regarding the molecular basis of the pathogen’s virulence. This may be a consequence of the diversity of *T. cruzi* strains used in different studies or the high level of variation in the genetic background of DTU isolates. Notably, strains used in previous studies were obtained from several hosts and different locations of the American continent [14,15]. Therefore, a suitable model for investigating the virulence of *T. cruzi* is needed to determine whether the parasite’s virulence could be related to the expression levels of a single virulence factor (or a group of the same) and/or reprogramming of biochemical processes linked to their expression, such as those associated with the generation of energy. Studies on *T. cruzi* cell lines derived from the same clone, but exhibiting different virulent phenotypes, could contribute to a better understanding of the molecular basis of such differences.

The goal of this study was to evaluate the contribution of previously identified virulence factors of *T. cruzi*, including Czp, TS, CRP, and Tc-85, to the pathogen’s virulence, using tissue culture-derived trypomastigotes (TCTs) derived from two genetically related *T. cruzi* cell lines that exhibit contrasting virulent and pathogenic characteristics. Applying a proteomics approach, this study elucidated the biological implications of the observed differences between the two cell lines and their potential relationship with the pathogen’s virulence.

## Material and methods

### Ethical approval

All experiments involving animals were performed in accordance with the guidelines of the Ethics Committee in Scientific Research (approval CEIC REV/2016) and Institutional Ethics Committee for Animal Experimentation (approval CEIC REV/2013) of the University of Antofagasta, Chile.

### Parasite cell lines and cell culture

The *T. cruzi* H510 C8C3 clone was used in all experiments [16]. It was generated from the *T. cruzi* House 510 strain, which was retrieved from Costa Rican *Triatoma dimidiata* [17]. Clonal cells were subjected to different treatments to generate the two cell lines used in the study. For the first treatment, clone cells were passaged in BALB/c mice once per week, for 30 years, following which the cells were subjected to periodic passages in BALB/c mice and Vero cells (*T. cruzi* H510 C8C3***hvir*** cell line). A second culture of clone cells was maintained in liver infusion tryptose (LIT) medium throughout the same 30 years, following which it was cultured cyclically in the LIT medium [18] and Vero cells (*T. cruzi* H510 C8C3***lvir*** cell line).

To obtain TCTs, Vero cells were infected with either of the two *T. cruzi* cell lines at a parasite: cell ratio of 5:1 for over 3 h. Cell cultures were then washed and incubated in RPMI 1640 medium supplemented with 2% foetal bovine serum [19]. After five days, the cell culture supernatants, highly enriched in TCTs, were collected by centrifugation and used for further experiments.

### Genotyping of T. cruzi C8C3hvir and C8C3lvir cell lines

DNA was isolated from TCTs of *T. cruzi* C8C3***hvir*** and C8C3***lvir*** cell lines using the Wizard Genomic DNA Purification Kit (Promega, Madison, WI, USA) according to the manufacturer’s instructions. Cell line genotyping was performed by PCR amplification of the 18S rRNA gene followed by DNA sequencing, as previously described [20]. Sequences were aligned using ClustalW software [21] and phylogenetic trees was constructed using MEGA X software [22] by the bootstrap method with 500 replicates. Maximum likelihood analysis was performed using a general time reversible substitution model. The topologies of the phylogenetic trees were confirmed using the neighbour-joining method with a *p*-distance matrix.

### TCT invasion assay

Neonatal mice were sacrificed and their myocardial cells were harvested according to a previously reported method [23]. The myocardial cells were then seeded at a density of 5 × 10^4^ cells/well using 4-well Lab-Tek Chamber Slides (Nunc, Thermo Fisher Scientific, Roskilde, Denmark).

The TCT invasion assay was conducted according to a previously reported method 19 using a parasite: cell ratio of 5:1. Following incubation for 3 h at 37 0C, the myocardial cells were washed thrice with phosphate-buffered saline (PBS), fixed with paraformaldehyde, and stained with propidium iodide. The number of parasites present in 500 cardiomyocytes was observed using a BX51 fluorescence microscope (Olympus Corporation, Tokyo, Japan).

### Parasite proliferation and metacyclogenesis

Epimastigotes from either the *T. cruzi* C8C3***hvir*** or C8C3***lvir*** cell line were cultured in LIT medium at a cell density of 1 × 10^6^ parasites/mL 18. The epimastigote growth curve was determined by counting the number of parasites every two days for 10 days. After culturing for seven days, metacyclogenesis was evaluated based on kinetoplast shapes and positions in smears treated with Giemsa stain. Additionally, metacyclogenesis was evaluated in the *T. cruzi* C8C3***hvir*** cell line cultured in LIT medium in the presence or absence of the cysteine protease inhibitor E-64d, according to a previously described method [24]. All experiments were performed in triplicate.

### TCT infection and parasitaemia curves

Four-to-six-week-old female BALB/c mice were inoculated with TCTs derived from either the *T. cruzi* C8C3***hvir*** or C8C3***lvir*** cell line through intraperitoneal injection (1 × 10^5^ parasites/mouse; 5 mice/treatment group). Presence of parasitaemia was recorded every second day, beginning the second day after inoculation, according to a previously described method [25]. The experimental series was suspended on day 18, as the C8C3***hvir***-infected mice began to perish on day 20. Mice with acute or chronic infection were sacrificed, followed by collection of the heart, lung, and liver, as well as tissue samples from the quadriceps muscle, for DNA isolation. The presence of *T. cruzi* in organs and/or tissues was detected by qPCR, according to a previously described method [26]. In order to demonstrate the infectivity of the C8C3***lvir*** cell line, mice were infected in groups of five as described above, and, thereafter, five days following infection, treated with three cycles of cyclophosphamide (Endoxan, Baxter Oncology, GmbH, Halle, Germany) at 50 mg/kg of body weight, according to a previously described method [27].

### DNA isolation and real-time quantitative PCR

Total genomic DNA was extracted from the organs and quadriceps muscle tissues of the infected mice using the Wizard Genomic DNA Purification Kit (Promega), according to the manufacturer’s instructions. The DNA concentration was quantified using an Infinite 200 PRO UV spectrophotometer (Tecan, Männedorf, Switzerland) and adjusted to 25 ng/μL.

Real-time PCR analysis was performed according to a previously described method [26]. The primers used were specific either for *T. cruzi* 195-bp repeat DNA sequences (TCZ-F: 5′-GCTCTTGCCCACAMGGGTGC-3′, where M = A or C and TCZ R: 5′-CCAAGCAGCGGATAGTTCAGG-3′), generating a 182-bp product, or for murine-specific tumour necrosis factor-α (TNF-α) (TNF-5241: 5′-TCCCTCTCATCAGTTCTATGGCCCA-3′ and TNF-5411: 5′-CAGCAAGCATCTATGCACTTAGACCCC-3′), amplifying an internal endogenous control gene to generate a 170-bp product [26]. The thermal cycling program carried out using the Eco Real-Time PCR system (Illumina Inc., San Diego, CA, USA) was as follows: denaturation at 95 °C for 10 min, followed by 40 cycles of 94 °C for 15 s, and 64.3 °C for 1 min, with fluorescence recording at 64.3 °C. Real-time amplification was followed by an initial denaturation step of 15 s at 95 °C, cooling to 60 °C for 1 min, and thereafter, a stepwise temperature increase of 0.3 °C/s from 60 to 95 °C. qPCR analysis of each sample included a standard curve and two negative controls. The negative controls comprised a reaction mixture containing *T*. *cruzi*- or murine-specific primers and from which parasite DNA was omitted or DNA from non-infected mouse tissues was included. All DNA samples were analysed in triplicate. The mean quantification values for *T. cruzi* DNA were normalized based on the data collected using murine-specific (TNF-α) primers and applying the following equation:

Normalized value = (mean of *T. cruzi* DNA value/mean of TNF-α DNA value) × 1000

where “1000” corresponds to the expected value of TNF-α derived from 30 mg of heart tissue. The amplification efficiencies were evaluated automatically by StepOne™ Software v2.0, using the following equation [28]:

Efficiency (*E*) = 10^(−1/slope)^

### Preparation of trypomastigote lysates and in-gel protein digestion

TCTs derived from *T. cruzi* C8C3***hvir*** or C8C3***lvir*** cell lines were recovered from the cell culture supernatant by centrifugation at 200 × *g* for 10 min, followed by a single wash in PBS and another round of centrifugation. Thereafter, the supernatant was discarded and the trypomastigote pellet quick-frozen in liquid nitrogen and stored at −80 °C until further use. Prior to use, the pellet, containing 5 × 10^7^ TCTs, was resuspended in 1.5 mL ice-cold purified water (Milli-Q) containing 1.5 mL trifluoroethanol (Sigma-Aldrich, Taufkirchen, Germany).

The TCTs protein extract was diluted with up to 50 µL sample buffer and then subjected to SDS-PAGE (1 mm thick, 4% stacking, and 10% resolving). The gel was stained with Coomassie blue dye, each run line was excised and cut into small pieces (2 mm × 2 mm), which were then transferred to microcentrifuge tubes [29]. Thereafter, the gel pieces were bleached in a solution of acetonitrile (ACN): water at a 1:1 ratio. Subsequently, the proteins in the gel pieces were reduced by incubating in 10 mM DTT for 1 h at 56 °C, and then alkylated by adding 10 mM iodoacetamide followed by incubation at 20 °C for 30 min in complete darkness. Thereafter, the proteins were subjected to *in situ* digestion using sequencing-grade trypsin (Promega, Madison, WI, USA), according to a previously described method [30]. Briefly, the gel pieces were shrunk by adding ACN, which was subsequently removed, and the pieces dried in a vacuum concentrator. The dried gel pieces were thereafter re-swollen in 100 mM Tris-HCl pH 8 containing 10 mM CaCl_2_. This was followed by the addition of trypsin (60 ng/µL) to the mixture, at a protein: enzyme ratio of 5:1 (w/w). The mixture was then maintained at 4 °C for 2 h followed by incubation at 37 °C for 12 h. Finally, trypsin digestion was interrupted by adding 1% trifluoroacetic acid (TFA). Thereafter, salts were removed using OMIX C18 pipette tips (Agilent Technologies, Santa Clara, CA, USA). The digested proteins were dried, solubilized in 10 µL 0.1% formic acid, and subjected to mass spectrometric analysis.

### Reverse phase-liquid chromatography-tandem mass spectrometry (RP-LC-MS/MS) analysis

The peptide mixture was analysed by RP-LC-MS/MS using the Easy-nLC II system combined with an ion trap LTQ-Orbitrap-Velos-Pro hybrid mass spectrometer (Thermo Fisher Scientific, Waltham, MA, USA). The peptides were concentrated (on-line) by reverse phase chromatography using a 0.1 mm × 20 mm C18 RP precolumn (Thermo Fisher Scientific), and then separated using a 0.075 mm × 250 mm C18 RP column (Thermo Fisher Scientific) operating at a flow rate of 0.3 μL/min. The peptides were eluted using the following dual gradient: 5–25% solvent B for 135 min, 25–40% solvent B for 45 min, 40–100% solvent B for 2 min, and 100% solvent B for 18 min (solvent A: 0.1% formic acid in water, solvent B: 0.1% formic acid and 80% ACN in water). Mass spectrometric analysis was conducted using electrospray ionization with a Nanobore stainless steel emitter (30 μm ID; Proxeon Biosciences, Roskilde, Denmark) at a spray voltage of 2.1 kV, an S-Lens of 60%, and resolution set to 30,000 [31].

Peptides were tracked in survey scans from 400 to 1600 amu (1 μscan), followed by 20 data-dependent MS/MS scans, using an isolation width of 2 u (mass-to-charge ratio units), normalized collision energy of 35%, and dynamic exclusion applied in 60 second periods. Unassigned and individually charged protonated ions were rejected by charge-state screening. Three replicates were generated for each *T. cruzi* cell line, producing a total of six runs.

### Protein data analysis

Raw data were imported for protein identification and quantification using the MaxQuant version 1.6.17 software [32]. Protein identification was performed using the Andromeda database search engine (19 October 2020 release) against UniProt entries for *T. cruzi (*TCC strain: 22,532 entries; Dm28c strain: 11,346 entries; and CL Brener strain: 19,242 entries). Carbamidomethylation of cysteine (57.021464 Da) was selected as a fixed modification, whereas oxidation of methionine (15.994915 Da) and *N*-terminal acetylation protein (42.010565 Da), were selected as variable modifications. Enzyme specificity was set to full trypsin with a maximum of two missed cleavages. The minimum peptide length was set to seven amino acids. Label-free Quantification (LFQ), using the “match-between-runs” feature in MaxQuant, was used to identify transfer between samples based on the retention time and accuracy of mass, with a match time window of 0.7 min and alignment time window of 20 min. Protein quantification was performed based on LFQ, and protein abundance was calculated based on the normalized spectral protein intensity (LFQ intensity).

Statistical analysis of the quantified proteins was performed using the Perseus version 1.6.14.0 software. Potential contaminants, and proteins identified only by site or in the reverse database, were excluded from further analysis. The LFQ intensity was log2-transformed, samples were categorized by group (C8C3***hvir*** or C8C3***lvir***), and a filter of two valid values in at least one group, was applied. Student’s *t*-test, followed by Benjamini-Hochberg correction (false discovery rate-adjusted p-value <0.05), was applied to the data. A significant difference in the expression of a protein between groups was considered to indicate a regulated protein. In addition, principal component analysis (PCA) was performed, and volcano plots and heatmaps constructed, to visualize protein expression patterns.

Gene ontology (GO) and enriched pathway analyses were performed using the TritrypDB platform (https://tritrypdb.org/tritrypdb/app), REVIGO algorithm (doi:10.1371/journal.pone.0021800) (http://revigo.irb.hr/), KEGG database (https://doi.org/10.1093/nar/gkv1070), MetaCyc database (doi: 10.1186/1471–2105-14-112), and the GOsummaries package in R (doi.org/10.12688/f1000research.6925.1). A q-value threshold of 0.05 (Benjamini-Hochberg corrected), was used. Analyses were performed separately for proteins identified as upregulated or downregulated between groups. Bioinformatics analysis was performed using the DAVID version 6.8 software (10.1038/nprot.2008.211) to assess clusters related to differentially regulated proteins. STRINGS tool (https://string-db.org/) (10.1093/nar/gky1131) and Omics Visualizer software (10.12688/f1000research.22280.2) were used to construct the protein interaction network, with *T. cruzi* selected as the organism and the confidence interaction set to 0.150. Sparse partial least squares discriminant analysis (PLS-DA) was performed to classify samples into known groups and identify key variables that drive discrimination using the mixOmics package in R (https://doi.org/10.1371/journal.pcbi.1005752).

### SDS-PAGE, 2-D gel electrophoresis, and immunoblotting

TCTs (C8C3***hvir*** or C8C3***lvir***) were lysed in 1 × PBS with 0.1% NP-40 containing cOmplete™ Mini EDTA-free Protease Inhibitor Cocktail (Thermo Fisher Scientific, Rockford, IL, USA) followed by determination of the protein concentration using the Bradford assay [33]. Proteins in the parasite extracts were separated by SDS-PAGE on a 12% acrylamide gel and transferred to nitrocellulose membranes using the Trans-Blot Turbo Transfer System (Bio-Rad Laboratories, Hercules, CA, USA). The membranes were incubated with an anti-*T. cruzi* Czp polyclonal antibody [34], mouse polyclonal antibody against recombinant CRP [35], or monoclonal antibody 39 against TS [36]. Anti-mouse or anti-rabbit IgG peroxidase-labelled antibodies were used as secondary antibodies. Immunoblot reactivity was detected by enhanced chemiluminescence assay (ECL; Thermo Fisher), and images were captured using the DNR Bio-Imaging System (DNR Bio-Imaging Systems, Ltd., Neve Yamin, Israel) with Gel-Capture software.

For 2-D gel electrophoresis, parasite proteins were precipitated with 10% trichloroacetic acid, and thereafter, 50 μg protein was placed onto 7 cm ReadyStrip IPG strips (pH 3–5, 4–7, and 3–10 non-linear; Bio-Rad Laboratories). The IPG strips were then rehydrated for 12 h at 20 °C and First-dimension isoelectric focusing (IEF) was performed using a Protean IEF Cell (Bio-Rad Laboratories) in a three-step procedure: 100 V for 1 h, 500 V for 1 h, and 4000 V until completion, for a total of 10,000 V h. Following IEF, the IPG strips were balanced and used for the second dimension. Thereafter, the IPG strips were loaded onto a 12% polyacrylamide gel and proteins resolved by SDS-PAGE. The proteins were then transferred onto nitrocellulose membranes for immunoblot detection using polyclonal antibodies against Czp and monoclonal antibodies against TS [37], Tc-85 [38], and CRP [39]. Images from the immunoblots were captured using the DNR Bio-Imaging System, as described above. To demonstrate that identical amounts of proteins were loaded in all lanes of each gel, the membranes were incubated with 50 mL stripping buffer (2% (w/v) SDS, 62.5 mM Tris, pH 6.7, and 100 mM 2-mercaptoethanol) for 1 h at 60°C in an orbital shaker. Thereafter, the membranes were washed six times with Tris buffer saline (TBS)-Tween 0.3% for 10 min each, blocked with 5% TBS-milk, and incubated with a mouse monoclonal antibody against β actin (Abcam, Cambridge, UK). ImageJ v2.0 software was used to perform densitometric analysis of the immunoblots, in which expression was normalized to that of β actin (loading control). In order to obtain consistent results, each experiment was performed at least in triplicate.

### Complement mediated-lysis (CML) assay

The CML assay was performed according to a previously described method [40]. Briefly, TCTs from either the *T. cruzi* C8C3***hvir*** or C8C3***lvir*** cell line (5.0 × 10^6^ parasites/100 μL serum-free RPMI) were incubated with either 100 μL 50% normal human serum or RPMI alone (as a negative control) for 30 min at 37 °C. Samples were then stained with trypan blue, and the percentage of lysed cells was determined in a Neubauer chamber using light microscopy. All experiments were performed in triplicate.

### Detection of enzymatic activities

**Figure 1.**
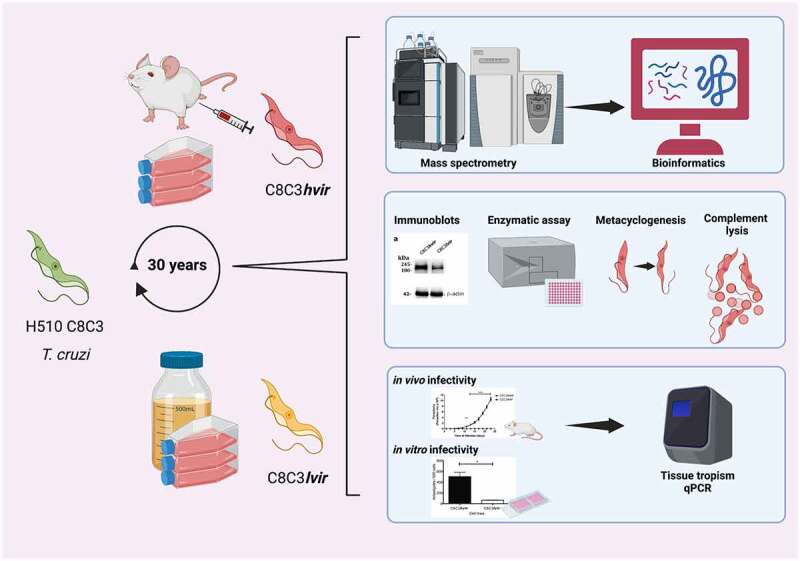
Experimental design to study C8C3*hvir* and C8C3*lvir Trypanosoma cruzi* cell lines and compare biological behaviour, proteomics profile, and virulence factor expression. *T. cruzi* tissue culture-derived trypomastigotes from both cell lines were used to infect BALB/c mice and evaluate parasitaemia curves and parasite loads in organs and tissues of acute and chronically infected mice. Metacyclogenesis was also studied. Trypomastigotes were lysed and supernatants were submitted to SDS-PAGE. Total proteins were in-gel digested by trypsin. Tryptic peptides were analysed by label-free quantitative mass spectrometry-based proteomics. The entire workflow was performed in triplicate for each *T. cruzi* cell line. Virulence factor protein expression was evaluated by immunoblotting. Cruzipain and transialidase enzymatic activity were measured. Created with Biorender.Com.

To investigate the Czp activity, TCTs or epimastigotes derived from *T. cruzi* cell lines (C8C3***hvir*** or C8C3***lvir***) were collected by centrifugation and lysed in PBS by sonication. The protein concentration of the soluble extract was determined according to a previously described method [33]. TCT proteins (100 μg) were immunoprecipitated using a polyclonal anti-Czp antibody, according to a previously described method [41]. Cysteine proteinase activity in the immunoprecipitate was detected by incubation with 10 μM Z-Phe-Arg-AMC (Sigma-Aldrich, St. Louis, MO, USA). Enzymatic activity was measured using an Infinite M200 PRO spectrofluorometer (Tecan) at an excitation wavelength of 353 nm and emission wavelength of 442 nm [42]. TS activity was determined according to a previously described method [43]. Briefly, parasite extracts were prepared and the TS activity of 50 µg total protein was measured using the Amplex™ Red Neuraminidase (Sialidase) Assay Kit (Thermo Fisher Scientific) according to the manufacturer’s instructions. Fluorescence was evaluated using the Infinite M200 PRO spectrofluorometer at an excitation wavelength of 540 nm and emission wavelength of 590 nm. The experimental approach developed in this study is illustrated in Figure 1.

### Statistical analysis

Statistical analysis was conducted using the GraphPad Prism version 9.0.1 (128) software (GraphPad Software, San Diego, Ca, USA). Student’s *t*-test and ANOVA were applied and significant differences, in infectivity or cell invasion capabilities, between *T. cruzi* C8C3***hvir*** and C8C3***lvir*** cell lines, were determined. *P*-values <0.05 were considered to be statistically significant. Survival analysis was calculated by the Kaplan-Meier method.

## Results

### T. cruzi C8C3hvir is highly infectious in mice and mouse cardiomyocytes

**Figure 2.**
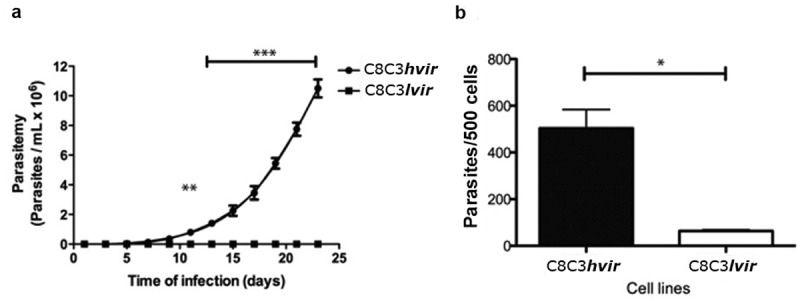
*In vitro* and *in vivo* infectivity of *T. cruzi* C8C3*hvir* and C8C3*lvir* cell lines. (a) Parasitemia curves of mice infected with trypomastigotes from either C8C3***hvir*** or C8C3***lvir***. Results are expressed as the mean ± SD and represent at least 3 experiments performed in triplicate. ** *P* < 0.01, *** *P* < 0.001; Two-way ANOVA. (b) Cardiomyocytes were infected with trypomastigotes from either C8C3***hvir*** or C8C3***lvir*** cell lines, using a parasite/cell ratio 5:1. After 3 h, cultures were washed three times with PBS. and stained with propidium iodide. Results are expressed as the mean ± SD of parasites/500 cells from three experiments performed in triplicate. * *P <* 0.01; Student’s *t*-test.

Parasitaemia curves were evaluated after mice were infected with TCTs derived from either the *T. cruzi* C8C3***hvir*** or C8C3***lvir*** cell lines (Figure 2a). C8C3***hvir***-infected mice exhibited high parasitaemia, reaching a peak on day 23 post-infection. Further, 80% of infected mice died between day 20 and 25 post-infection (Figure S1A). In contrast, C8C3***lvir-***infected mice displayed very low or subpatent parasitaemia during the experiment and survived for more than 60 days post-infection (Figure 2a). However, mice immunosuppressed by cyclophosphamide treatment exhibited moderate parasitaemia compared to the control group, which exhibited subpatent parasitaemia (Figure S1B). The results of the *in vitro* cell invasion assay demonstrated that TCTs derived from the C8C3***hvir*** cell line were three to five times more infectious in mouse cardiomyocytes than those from the C8C3***lvir*** cell line (Figure 2b).

### Mice infected with T. cruzi C8C3hvir display high parasite loads in muscle and heart tissues

**Figure 3.**
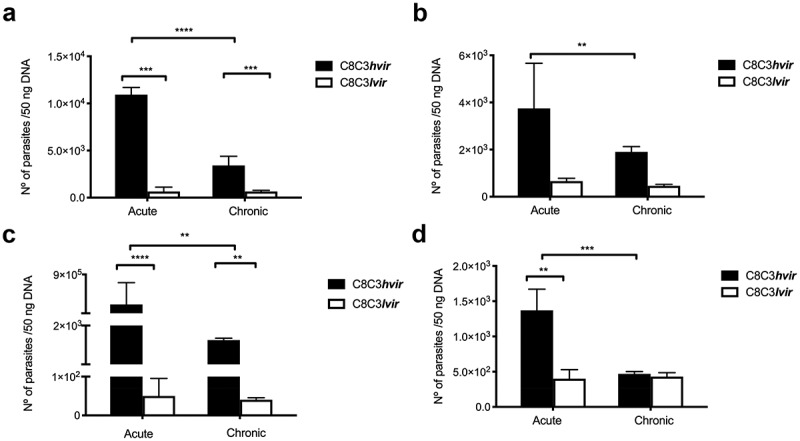
Organ and tissue parasitic load of mice infected with either *T. cruzi* C8C3*hvir* or C8C3*lvir* cell lines. Acutely or chronically infected mice were sacrificed and parasite load (parasites/50 ng DNA) was determined by qPCR in (a) heart, (b) liver, (c) quadriceps muscle, and (d) lung. The limit of detection was 0.1 parasite equivalent/50 ng DNA. Results are expressed as the mean ± SD and represent three experiments performed in triplicate. * *P* <0,01, ** *P <* 0,01 *** *P* < 0,001; Student’s *t*-test.

The parasite load in heart tissues collected from mice acutely infected with C8C3***hvir*** was nine times that in heart tissues of those acutely infected with C8C3***lvir*** (Figure 3a). Similar results were obtained in case of chronically infected mice, wherein the heart tissues of C8C3***hvir***-infected mice had a higher parasite load than those of C8C3***lvir***-infected mice (Figure 3a). Similarly, the parasite load was significantly higher in the liver tissues of mice acutely infected with C8C3***hvir***, being six times that in the liver tissues of C8C3***lvir***-infected mice (Figure 3b). Similar results were obtained in case of chronically infected mice, wherein the liver tissues of C8C3***hvir***-infected mice had a higher parasite load than those of C8C3***lvir***-infected mice (Figure 3b). Notably, the parasite load in the muscle tissues of mice acutely infected with C8C3***hvir*** was highest amongst all tissues investigated, and was also higher than that in muscle tissues of C8C3***lvir***-infected mice, reaching levels of 4.0 × 10^5^ parasites/ng DNA. Similar results were observed in case of muscle tissues of chronically infected mice wherein an increased parasite load was observed in the quadriceps tissues of mice infected with C8C3***hvir***. (Figure 3c). The parasite load in the lung tissues of mice acutely infected with C8C3***hvir*** was three times that in the lungs of C8C3***lvir***-infected mice. However, no differences were observed between the parasite load in the lung tissues of mice chronically infected with C8C3***hvir*** or C8C3***lvir*** (Figure 3d).

### Epimastigotes derived from T. cruzi C8C3hvir cell line display a high rate of metacyclogenesis

**Figure 4.**
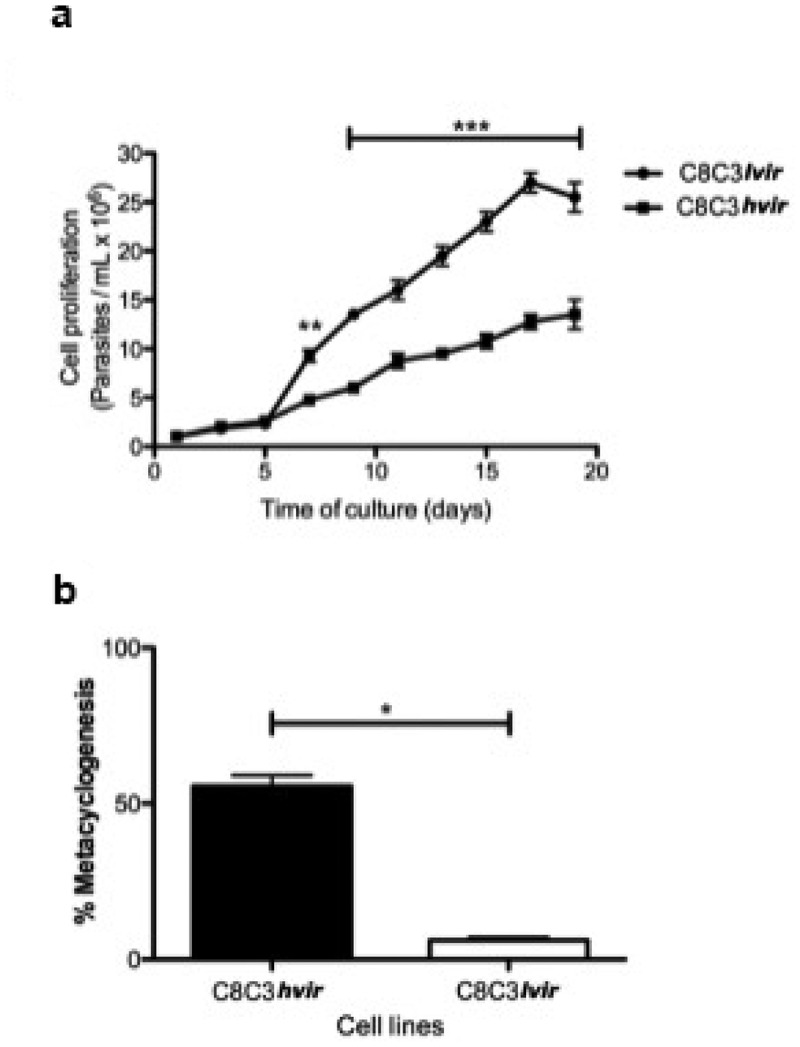
Epimastigote proliferation curve and metacyclogenesis of *T. cruzi* C8C3*hvir* and C8C3*lvir* cell lines. (a) Epimastigote growth. Results are expressed as the mean ± SD and represent at least three experiments performed in triplicate. ***P < 0.001; Two-way ANOVA. (b) Epimastigote differentiation into metacyclic trypomastigotes. Results are expressed as the mean ± SD and represent at least three experiments performed in triplicate. *P < 0.05; Student’s *t*-test.

Epimastigotes derived from the C8C3***lvir*** cell line displayed a higher capacity to proliferate in LIT medium than those derived from the C8C3***hvir*** cell line, which replicated at a slower rate. These results suggested that the rapid growth of epimastigotes derived from the C8C3***lvir*** cell line was due to their better adaptation to LIT medium (Figure 4a). In contrast, C8C3***hvir*** cells displayed a higher epimastigotes to MTs differentiation rate (approximately 50%) after culturing for 10 days than C8C3***lvir*** cells, which showed a very low differentiation rate (approximately 8%) (Figure 4b). Notably, the ability of different species of triatomines to generate metacyclic forms is of great importance in the transmission of parasitic infections [44].

### T. cruzi clone H510 C8C3 belongs to DTU TcIa

The typing of the *T. cruzi* cell lines was performed by PCR amplification followed by DNA sequencing. PCR amplification of mini-exon repetitive region units, which are DTU TcIa markers, confirmed that this region is conserved and that clone H510 C8C3 belongs to the DTU TcI. Amplification of the V7/V8 region of the ribosomal subunit gene (SSU RNA) [45] further supported these results. The phylogenetic tree depicted that both C8C3***hvir*** and C8C3***lvir*** were clustered within the TcI branch (Figure S2). As expected, the percentage identity between the two variants was high (99%). These results confirmed that C8C3***hvir*** and C8C3***lvir*** are genetically related and share a common origin (Figure S2).

### Comparative proteomic analysis of T. cruzi C8C3hvir and C8C3lvir cell lines

**Figure 5.**
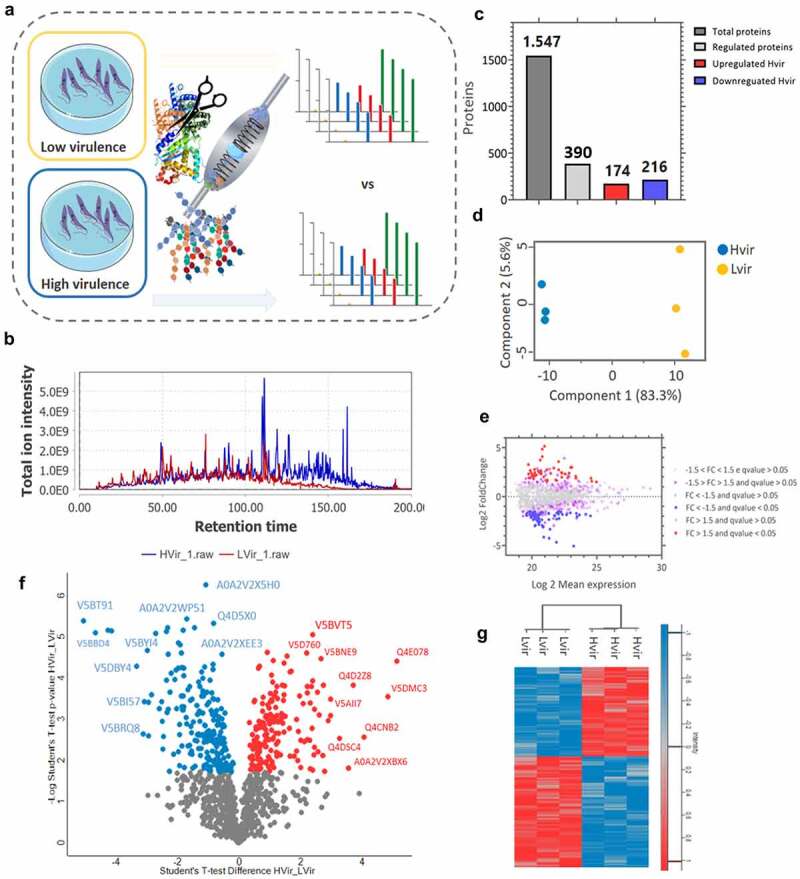
Workflow of proteomics analysis. Low-virulence (C8C3***lvir***, n = 3) and high-virulence (C8C3***hvir***, n = 3) cell lines were analysed by nLC-MS/MS and the data were mapped using concatenated databases for the Dm28c, TCC, and CL Brener *T. cruzi* strains. (a) Chromatographic elution profile of the peptides. (b) Identification of differentially regulated proteins. (c) Principal component analysis of differentially regulated proteins. (d) MAplot of differentially regulated proteins. (e) Volcano plot of differentially regulated proteins (F). Heatmap of differentially regulated proteins. The red and blue colours correspond with upregulated and downregulated proteins, respectively. The grey colour indicates that the protein was not differentially expressed.

Protein extracts of C8C3***hvir*** as well as C8C3***lvir*** cell lines were subjected to mass spectrometry, and the data were mapped (using MaxQuant software) using concatenated databases for the Dm28c, TCC, and CL Brener *T. cruzi* strains (Figure 5a). The chromatographic elution profiles of peptides derived from both the *T. cruzi* cell lines are shown in Figure 5b. A summary of the identified proteins is shown in Figure 5c. A total of 1,547 proteins were identified, of which 390 were considered to be regulated. Of these, 174 proteins were upregulated in the C8C3***hvir*** cell line whereas 216 were downregulated in the C8C3***lvir*** cell line (Figure 5c). The PCA results indicated differences between the two parasite cell lines (Figure 5d), with a clear demarcation between two distinct groups, based on the proteome profile. The results of the MAplot and volcano plot of protein expression are shown in Figure 5(e,F), respectively. Collectively, these results indicated that the proteins with the greatest variation in expression between the two parasite cell lines included prostaglandin F2α synthase, phosphate dikinase, chaperonin HSP60, ribosomal protein L24, and myosin heavy chain. A heatmap of the differentially regulated proteins revealed the presence of two inversely regulated protein clusters in the C8C3***lvir*** and C8C3***hvir*** cell lines.

**Figure 6.**
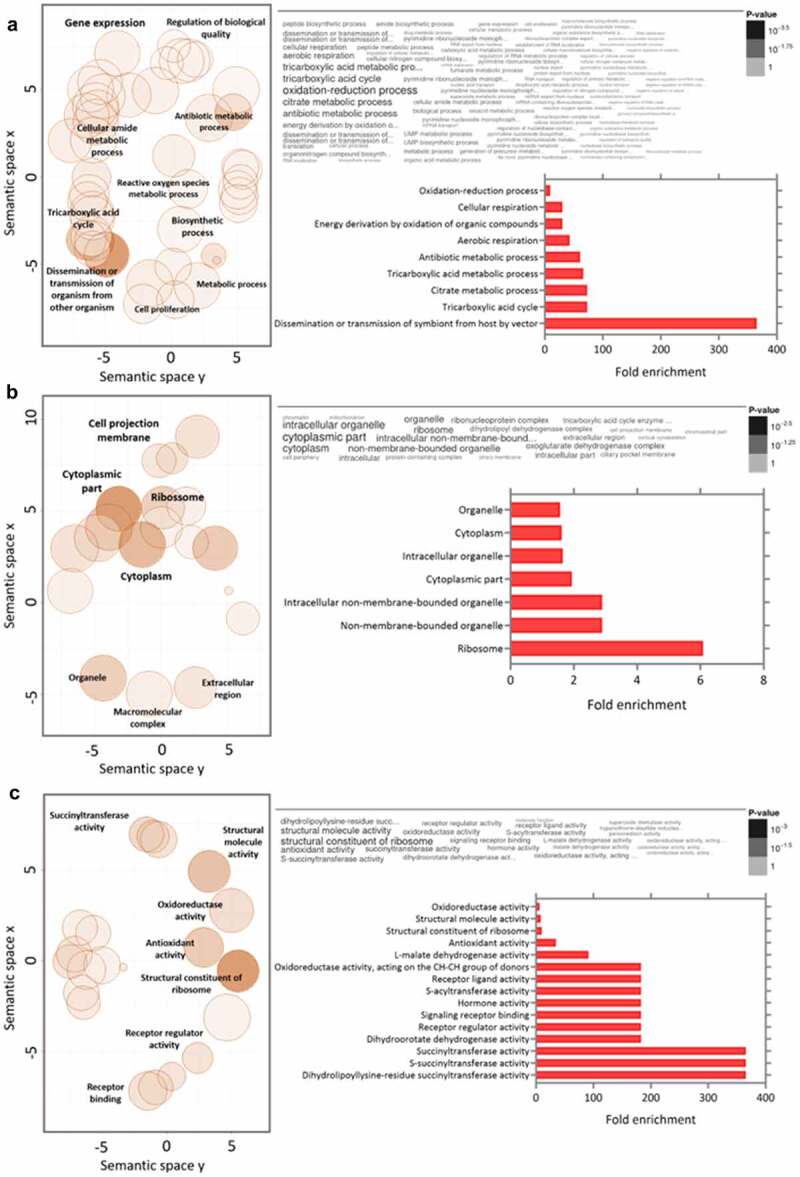
Gene ontology analysis combined with quantitative data for differentially expressed proteins in *T. cruzi* C8C3*hvir* cell line. Upregulated proteins in C8C3***hvir*** associated with (a) biological processes, (b) cellular components, and (c) molecular functions. Only ontologies with q-value ≤0.05 are presented (Benjamini-Hochberg corrected). The word cloud diagram indicates terms in size and colour proportional to -log(*p*-value). The histogram indicates the fold enrichment of each ontology term, presenting overrepresented terms in comparison to a gene background. The bubble chart shows the geometric position of each identified ontology term, illustrating the proximity between them.

GO analysis of the upregulated proteins in the C8C3***hvir*** cell line revealed enrichment of biological processes (BPs) proteins such as those related to dissemination or transmission of symbionts by a vector, followed by the tricarboxylic acid (TCA) cycle and citrate metabolism proteins (Figure 6a). Enriched cellular components (CCs) included ribosomal proteins, non-membrane-bound organelles, and intracellular non-membrane-bound organelles (Figure 6b, Table S1). Finally, molecular functions (MFs) proteins that were enriched included dihydrolipoyllysine-residue, succinyl transferase, and S-succinyl transferase activities proteins (Figure 6c, Table S2).

**Figure 7.**
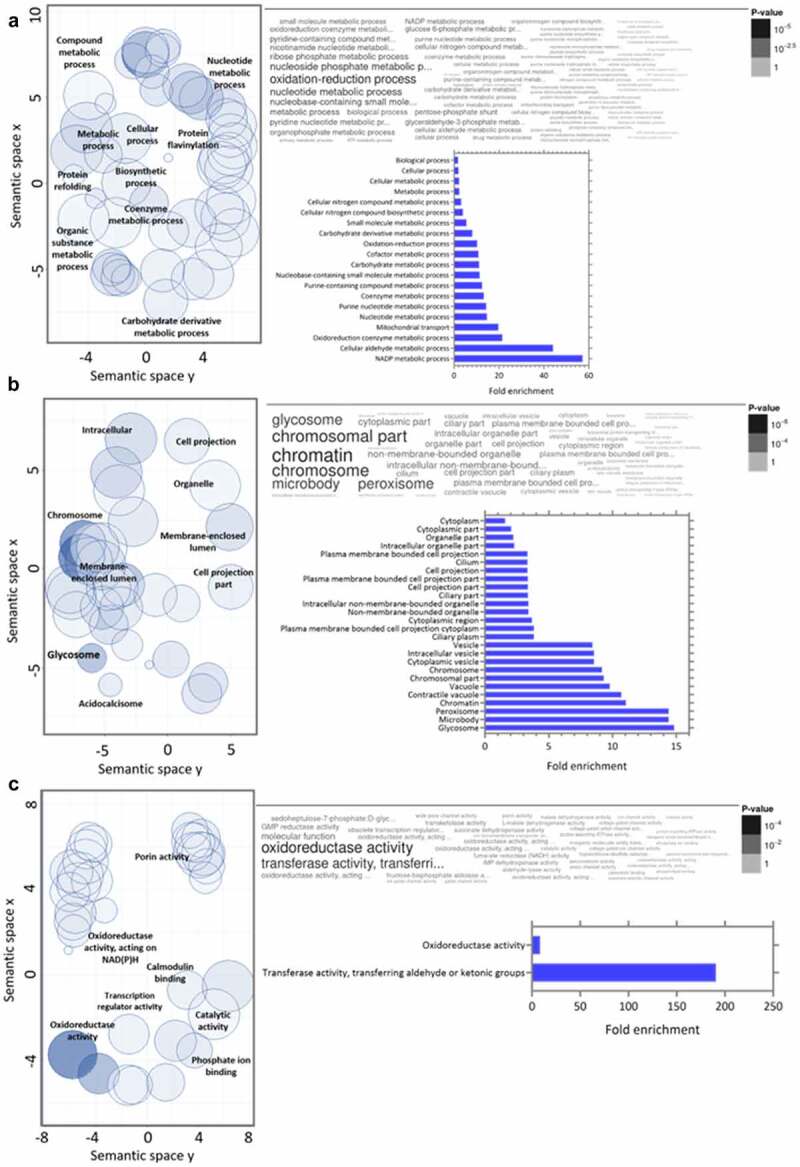
Gene ontology analysis combined with quantitative data for differentially expressed proteins in *T. cruzi* C8C3*lvir* cell line. Downregulated proteins in C8C3***lvir*** associated with (a) biological processes, (b) cellular components, and (c) molecular functions. Only ontologies with q-value ≤0.05 are presented (Benjamini-Hochberg corrected). The word cloud diagram indicates terms in size and colour proportional to -log(*p*-value). The histogram indicates the fold enrichment of each ontology term, presenting overrepresented terms in comparison to a gene background. The bubble chart shows the geometric position of each identified ontology, illustrating the proximity between them.

**Figure 8.**
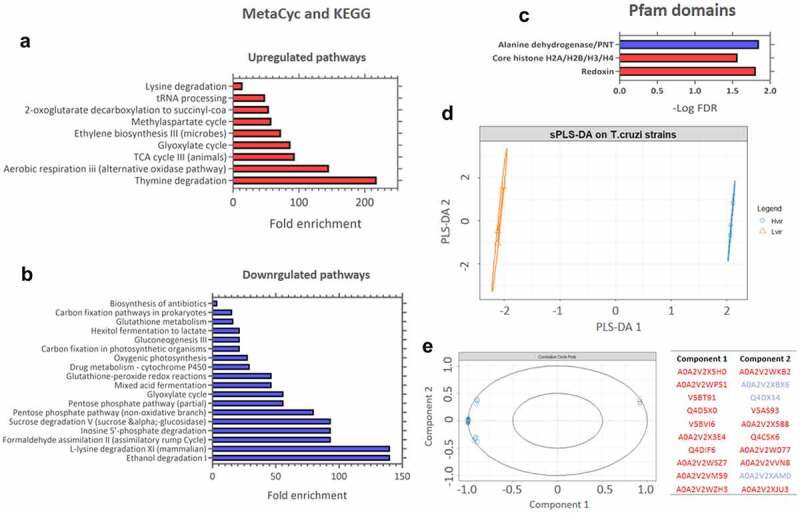
KEGG pathway enrichment analysis of upregulated and downregulated proteins in *T. cruzi* C8C3*hvir* cell line. Pfam and Interpro domains enriched in (a) upregulated and (b) downregulated proteins. Only pathways with a q-value ≤0.05 (Benjamini-Hochberg corrected) are presented. (c) Results of the sparse PLS-DA analysis. (d) Proteins are classified into known groups. (e) Key variables that drive the discrimination. The top 20 variables that contribute to components 1 and 2 are inside the circle (dim = 1). The red and blue colours correspond to upregulated and downregulated proteins, respectively.

GO analysis of downregulated proteins in the C8C3***lvir*** cell line revealed that BPs enriched included those of Nicotinamide adenine dinucleotide (NAD) metabolism, the cellular aldehyde process, and oxidoreduction coenzyme metabolism (Figure 7a). Enriched CCs included glycosomes, microbodies, and peroxisomes (Figure 7b). Finally, amongst MFs proteins, transferase activity proteins, which transfer aldehyde or keto groups, and oxidoreductase activity proteins, were enriched (Figure 7c). Amongst regulated pathways, proteins for degradation pathways for ethanol, L-lysine, inosine 5’-phosphate, and sucrose (Figure 8b) were enriched in the C8C3***lvir*** cell line, whereas proteins of pathways associated with thymine degradation, aerobic respiration, and the TCA cycle were enriched in the C8C3***hvir*** cell line (Figure 8a).

To verify the proteins that contributed most to virulence distinction between the two parasite cell lines, a sparse PLS-DA was performed (Figure 8d). C8C3***hvir*** and C8C3***lvir*** cell lines were most differentiated by the proteins shown in Figure 8e, suggesting that the proteins upregulated in the C8C3***hvir*** cell line contributed most to the virulence difference between the two cell lines.

### Trypomastigotes derived from the T. cruzi C8C3hvir cell line display higher expression of virulence factors and rate of metacyclogenesis than those derived from the T. cruzi C8C3lvir cell line

**Figure 9.**
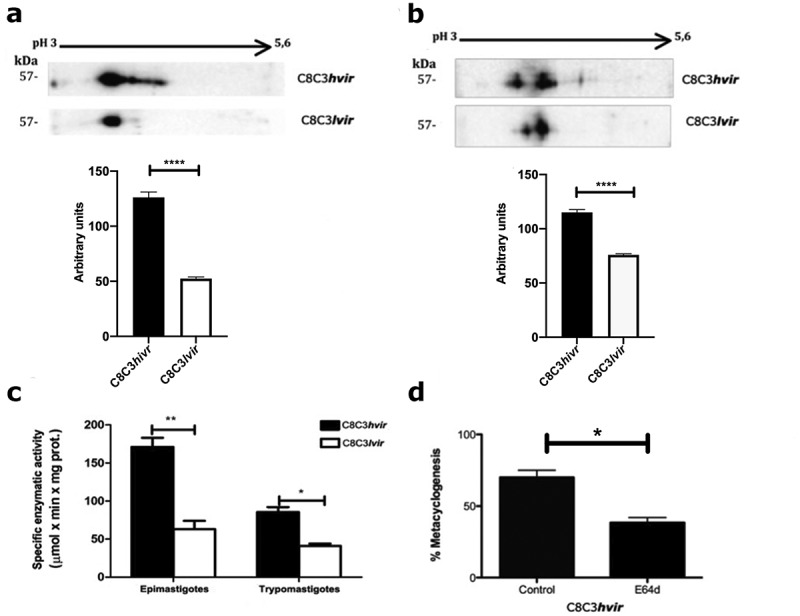
Cruzipain expression and metacyclogenesis in *T. cruzi* C8C3*hvir* and C8C3*lvir* cell lines. Two-dimensional electrophoresis results for cruzipain expression in (a) trypomastigotes and (b) epimastigotes from *T. cruzi* C8C3***hvir*** and C8C3***lvi*****r** cell lines. Densitometric analysis of immunoblots were performed using β actin as loading control. Bars are represented as the mean ± SEM of the least three independent experiments. ****P < 0.0001 vs corresponding control; Student’s *t*-test. (c) Cysteine proteinase activity of both cell lines. * *P* <0.5, **P < 0.05; Two-way ANOVA. (d) Metacyclogenesis in the presence or absence of cysteine proteinase inhibitor E64d, noted as percentage of metacyclic trypomastigotes.

TCTs (Figure 9a) and epimastigotes (Figure 9b) derived from the C8C3***hvir*** cell line displayed higher levels of Czp expression than those derived from the C8C3***lvir*** cell line. Additionally, trypomastigote Czp-specific enzymatic activity was higher in the C8C3***hvir*** cell line than in the C8C3***lvir*** cell line, as expected (Figure 9c). Since Czp is involved in *T. cruzi* metacyclogenesis [46,47], effects of the cysteine protease inhibitor, E-64d, on metacyclogenesis, were evaluated for the C8C3***hvir*** cell line. The results indicated that metacyclogenesis was strongly inhibited when parasite cells were co-cultured with E-64d, suggesting that Czp is involved in metacyclogenesis in the C8C3***hvir*** cell line (Figure 9d).

**Figure 10.**
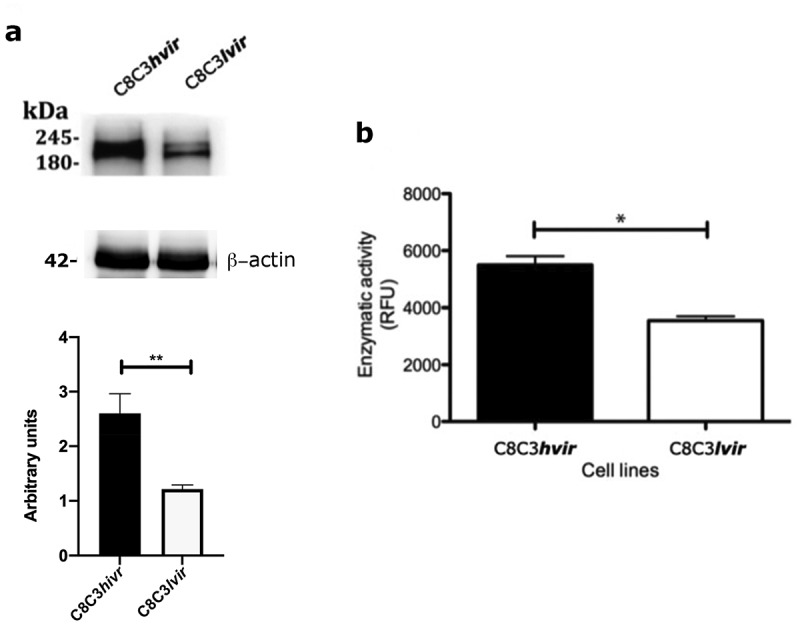
Trans-sialidase expression in *T. cruzi* C8C3*hvir* and C8C3*lvir* cell lines. (A) Trans-sialidase expressionin C8C3***hvir*** and C8C3***lvir***cell lines. Densitometric analysis of immunoblots were performed using b actin as loading control. The results are represented as the mean + SEM of five independent experiments. ** *P *= 0.029 vs corresponding control; Student’s t-test. (B) Trans-sialidase activity of trypomastigotes from C8C3***hvir*** and C8C3***lvir*** *T. cruzi* cell lines. All assays were performed in triplicate. * *P < *0.01;Student’s t-test.

TCTs derived from the C8C3***hvir*** cell line displayed higher TS expression than those derived from the C8C3***lvir*** cell line (Figure 10a). This was corroborated by assessment of TS enzymatic activity, wherein higher level of TS enzymatic activity was observed in the C8C3***hvir*** cell line than in C8C3***lvir*** cell line (Figure 10b).

**Figure 11.**
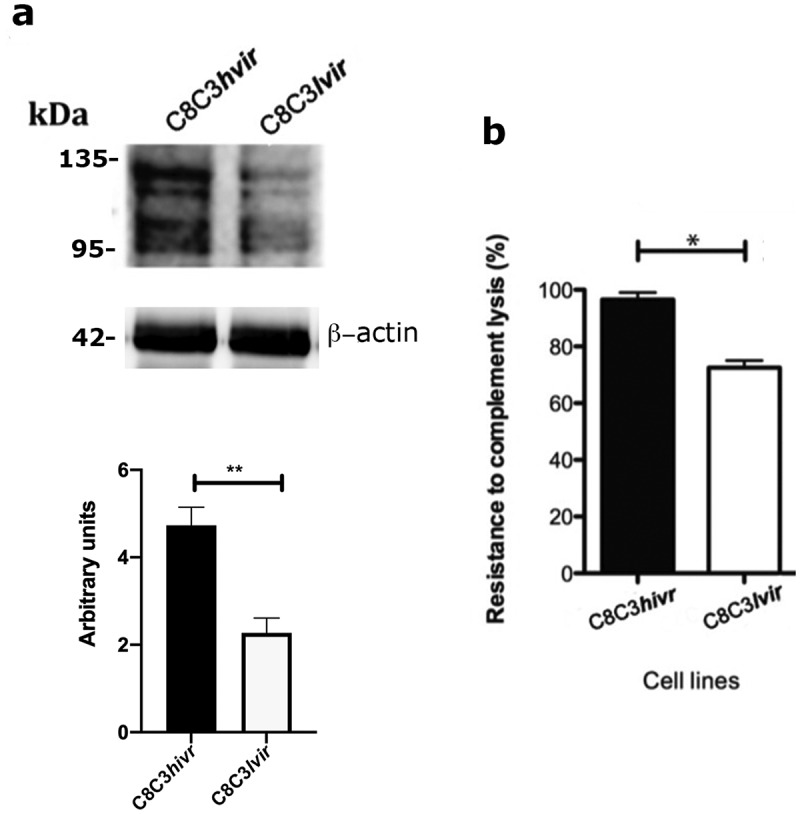
Complement regulatory protein (CRP) expression and complement-mediated lysis resistance in *T. cruzi* C8C3*hvir* and C8C3*lvir* cell lines. (a) CRP expression in trypomastigotes from C8C3***hvir*** and C8C3***lvir*** cell lines. Densitometric analysis of immunoblots were performed using β actin as loading control. =the results are represented by the mean ± SEM of five independent experiments. ** *P* = 0.0014 vs corresponding control; Student’s *t*-test. (b) Susceptibility of trypomastigotes from C8C3***hvir*** and C8C3***lvir*** cell lines to complement-mediated lysis. The results are expressed as the mean ± SD of experiments performed in triplicate. * *P <* 0.01; Student’s *t*-test.

As expected, CRP had a higher level of expression in the C8C3***hvir*** cell line than in the C8C3***lvir*** cell line (Figure 11a). This higher level of CRP expression aligned with the higher resistance of the C8C3***hvir*** cell line to CML (Figure 11b). Finally, expression levels of Tc-85 and sialylated epitopes were higher in the C8C3***hvir*** cell line than in the C8C3***lvir*** cell line (Figure 3S).

## Discussion

Studies conducted over the last few years have deepened our understanding of *T. cruzi* virulence [4,5]. Moreover, efforts have been made to establish the molecular basis for the virulence of MTs [3] or bloodstream trypomastigotes using clones of same *T. cruzi* strains [13]. Although several studies have reported various aspects of *T. cruzi* proteomics [48–53], only a few have compared the global protein expression in different strains or zymodemes [54–57]. Meanwhile, *T. cruzi* virulence has not been assessed from a proteomics perspective.

In this study, a combination of biological and proteomics approaches was employed to analyse high- as well as low-virulence cell lines (C8C3***hvir*** and C8C3***lvir***) derived from the *T. cruzi* H510 C8C3 clone [58]. Infection studies, performed both *in vivo* and *in vitro*, demonstrated that the C8C3***hvir*** and C8C3***lvir*** cell lines differed in their infectivity behaviour. Therefore, their proteomes were compared using GO, metabolism enrichment analysis, and PCA, which revealed differences between the two parasite cell lines (Figure 5d).

Differentially enriched proteins related to BPs were those associated with the dissemination or transmission of symbionts by a vector, the TCA cycle, and citrate metabolism (Figure 6a). The putative ADP ribosylation factor 3 (ARF3), in vector-transmitted symbiotic pathogens, has been shown to play a role in eukaryotic cellular trafficking and actin remodelling [59]. ARF3 has been described in trypanosomatids, including TcARf1 of *T. cruzi* [60], but its biological role remains unknown. However, ARF3 is essential for flagellar integrity in *Leishmania* [61]. Thus, presence of a healthy flagellum could be a prerequisite for *T. cruzi* virulence, considering that novel functions, beyond energetics and motility, have been discovered for kinetoplastid flagella [62]. Amongst these is the flagellar function that enables parasites to sense the presence of mammalian cells and modify their motility, thereby increasing cell invasion efficiency [63].

Several pieces of evidence obtained in the current study suggested that metabolic pathways involved in energy production, such as the TCA cycle, may be related to the higher virulence of C8C3***hvir*** compared with C8C3***lvir***. Indeed, among BPs, the TCA cycle and citrate metabolism proteins appeared to be upregulated in the virulent cell line. Likewise, genome-scale metabolic models have revealed that a functional TCA cycle is required by trypomastigotes to supply energy for flagellar activity [64]. These observations are in accordance with the study by Schenkman *et al* [65], in which attachment to mammalian cells required energy expenditure by *T. cruzi* trypomastigotes [65]. In contrast, positive regulation of the TCA cycle could be related to the eventual use of certain TCA cycle enzymes for the metabolic processing of carbon or other free energy sources, including amino acids such as glutamate and proline [66]. Notably, proline plays a critical role in *T. cruzi* cell invasion [67,68]. Furthermore, TCA cycle products, such as succinate and acetyl coenzyme A (acetyl CoA), may play an important role in *T. cruzi* virulence. Bloodstream trypomastigotes produce organic acids during glucose catabolism [69–71], with succinate being the major catabolite [72]. In eukaryotes, elevated levels of cytosolic succinate induce epigenetic alterations, production of mitochondrial reactive oxygen species (ROS), and protein succinylation of lysines (Ksucc) [73–75]. Although succinylation has not been described in *T. cruzi*, histone lysine succinylation is critical not only for the growth and differentiation of the human pathogenic protozoa *Toxoplasma gondii*, but also for the pathogen’s stage-specific adaptations to different environments during the transition between hosts [76]. Therefore, the findings of the current study strongly suggest that energy pathways such as the TCA cycle are involved in the virulence machinery of *T. cruzi*, particularly considering that the proteins of the pathway were upregulated in the C8C3***hvir*** cell line and downregulated in the C8C3***lvir*** cell line.

In this study, peroxisomes, microbodies, and glycosomes were comparatively more downregulated in the C8C3***hvir*** cell line than in the C8C3***lvir*** cell line. From an evolutionary point of view, parasite glycosomes, wherein glycolysis enzymes are compartmentalized [77], are the most divergent type of peroxisomes [76]. Prior to *T. cruzi* invasion, important changes reportedly occur in the trypomastigote phosphoproteome, following contact with the extracellular matrix [78]. These changes include a decrease or increase in the phosphorylation of several glycosomal enzymes, which appear to be related to reduced levels of several carbon metabolism intermediates. These observations indicate that contact with the extracellular matrix may significantly modulate the glycolytic pathway in infective forms of *T. cruzi* [78].

Differentially enriched proteins related to cellular components (CCs) were those associated with ribosomal proteins, non-membrane-bound organelles, and intracellular non-membrane-bound organelles (Figure 6b). Previous studies on fibroblasts have shown that TCTs, derived from *T. cruzi* that were recently released from cells, showed low infectivity and required an extracellular maturation process to become highly infective [79,80]. Treatment of trypomastigotes with protein synthesis inhibitors [79,81] markedly reduced parasitic invasiveness, suggesting that protein synthesis is crucial for adhesion and cell invasion of *T. cruzi* [81]. Amongst the newly synthesized proteins, those expressed on the surface of the cell membrane as well as those loaded on extracellular vesicles, participate in parasite adhesion and invasion, thus increasing heart parasitism [82–85]. In addition, protein expression in *T. cruzi* TcI strains is reportedly increased to stimulate higher motility as well as neutralize macrophage-released products such as free radicals in order to improve the odds of the pathogen’s survival in the mammalian host [86].

Finally, differentially enriched proteins related to MFs were those associated with various succinyl transferase activities (Figure 6c). One of the first steps of the TCA cycle involves the production of acetyl CoA from pyruvate, generated during glycolysis, by the mitochondrial pyruvate dehydrogenase complex (PDC), which then leads to the synthesis of fatty acids [87] and sterols [88]. PDC [89] is a multi-enzymatic complex composed of several copies of E1 (subunits α and β pyruvate dehydrogenase), E2 (dihydrolipoyl transacetylase), and E3 (dihydrolipoyl dehydrogenase) subunits, in addition to the E3-binding protein [90]. Several enzyme complexes, such as PDC, require lipoic acid as a prosthetic group. A subunit of lipoylated PDC E2 was previously detected in *T. cruzi* via immunoblotting [91]. These observations suggest that, in addition to the roles played by fatty acids, as energy sources or signalling molecules [87] as well as for sterol enrichment in the flagellum during the formation of lipid rafts involved in cell invasion or host immune response evasion [92], biochemical pathways such as the TCA cycle are critical for trypomastigote virulence.

KEGG pathway enrichment analysis revealed that proteins related to thymine degradation were highly upregulated in the C8C3***hvir*** cell line, followed by those involved in aerobic respiration (oxidase pathways) and the TCA cycle. A previous study reported that a glucosylated thymine DNA base, named base J [93], plays a role in the virulence of *T. cruzi* presumably because its reduction or loss led to an increase in the rate of Pol II transcription as well as higher expression of virulence-related genes, that resulted in increased parasitic invasiveness [94]. The upregulation of proteins associated with bioenergetic pathways, such as the TCA cycle, as well as oxidative pathways, supports the involvement of energy production processes in the virulence of trypomastigotes of *T. cruzi*.

During *T. cruzi* invasion, the host’s innate and adaptive immune responses lead to the release of ROS and reactive nitrogen species (RNS) [95]. Trypomastigotes need to resist deleterious attacks from non-immune cells because a broad range of ROS and RNS are synthesized as a result of metabolism under aerobic conditions [95]. *T. cruzi* reportedly possesses an arsenal of antioxidative enzymes, such as superoxide dismutases, tryparedoxin peroxidases, and peroxidases, to reduce the levels of ROS and RNS [96–104]. Many of these enzymes appear to be upregulated in the infective stages of *T. cruzi* [86,96,105–107]. In the present study, among Pfam domains, redoxins were enriched in the C8C3***hvir*** cell line relative to the C8C3***lvir*** cell line, suggesting that these may be associated with parasite virulence. According to Zago *et al* [86], *T. cruzi* TcI strains, such as C8C3***hvir***, employ several antioxidant enzymes to promote the pathogen’s development in macrophages thus causing damage to the infected mice.

A recent study reported that *T. cruzi* mitochondrial calcium-sensitive pyruvate dehydrogenase phosphatase (TcPDP) is required for energy metabolism, proliferation, cell differentiation, and invasiveness of *T. cruzi* [108]. Therein, it was shown that the cell invasion abilities of TcPDP-knockout trypomastigotes was impaired compared to that of wild-type trypomastigotes. Considering that TcPDP dephosphorylates TcPDH, a critical component of the TCA cycle, stimulation of energy metabolism through TCA cycle activation may be essential for parasite invasion [108]. On the other hand, pyruvate, the final product of the glycolytic pathway, needs to be actively transported to the mitochondrial matrix. Pyruvate transporter subunits have been reported to be required for pyruvate-driven respiration, cell invasion, and amastigote proliferation in *T. cruzi* [109]. Further, genetic suppression of mitochondrial pyruvate transporter subunits, MPC1 and MPC2, significantly reduces *T. cruzi* invasion and amastigote proliferation [109]. The ablation of any of the components of this bioenergetic process resulted in impaired infectivity and virulence [109]. The results of these studies suggest that compounds that cause mitochondrial inefficiency may be promising trypanocidal drug candidates [110]. Similarly, chemotherapeutic interventions targeting bioenergetic processes [111,112], redoxins [105], and ribosomes, have been proposed [113–115].

Differences between the C8C3***hvir*** and C8C3***lvir*** cell lines at the proteomics level should be reflected in the differences in their virulence factor expression, considering that the increased virulence of the C8C3***hvir*** cell line, in addition to differential expression of some well-known virulence factors, could be related to its greater ability to invade host cells. In the current study, western blotting demonstrated that Czp, Tc-85, TS, and CRP were expressed in both cell lines, but the C8C3***hvir*** cell line displayed higher levels of these virulence factors than the C8C3***lvir*** cell line. Moreover, the expression of sialylated epitopes was higher in the C8C3***hvir*** cell line than in the C8C3***lvir*** cell line (Figure S3). Various reports have suggested that Czp and members of the gp85/TS protein family are crucial for *T. cruzi v*irulence [4]. These proteins participate in parasite infectivity and intracellular growth [116–120]. TS also participates in host immune evasion [116–119], and CRPs, which are present only on trypomastigotes, block complement system activation [120]. In addition, the complement system modulates the infectivity of susceptible *T. cruzi* TcI strains [121]. Thus, CRP expression appears to be closely related to *T. cruzi* virulence [10], which was supported in the current study. Considering that the host complement system functions as a natural defence mechanism that *T. cruzi* needs to resist in order to infect the mammalian host [122], one of the possible explanations for the differences in virulence observed in the current study may be the increased ability of the C8C3***hvir*** cells to invade the host cells and resist the complement system. Increased expression of Czp, TS, CRP (Figure 9a, Figure 10a and Figure 11a), and Tc-85 (Figure S3) in the C8C3***hvir*** cell line compared with the C8C3***lvir*** cell line suggests a greater invasive capacity of the virulent cell line. The results also suggest a greater affinity of the C8C3***hvir*** cells for cardiac tissue, which may result from the high expression of glycoproteins such as Tc-85 which contains the FLY motif at its C-terminus [123]. FLY promotes parasite cell adhesion to endothelial cells, thus contributing to *T. cruzi* myocardial affinity [123]. In the present study, heart tissues from C8C3***hvir***-infected mice displayed a high parasite load, which was consistent with previously reported observations. This observation also supports the involvement of *T. cruzi* TcI strains in cardiac forms of Chagas disease, suggesting that some TcI strains are more virulent than other DTUs [124] and may be implicated in the progression of cardiac forms of Chagas disease, including the fatal acute Chagas disease [124,125]. TcI is also the most predominant genotype found in blood as well as heart biopsy samples of patients with Chagas cardiomyopathy [124,126–128].

Despite this body of evidence, it remains unclear as to why the expression of factors such as Czp, TS, CRP, and Tc-85 results in a more virulent parasite. A previous study reported that inhibition of each of these virulence factors reduces the levels of parasitaemia and cell invasion, but does not completely abolish infection [58]. The results therein suggested that *T. cruzi* trypomastigotes express a “virulent genetic program,” which includes upregulation of proteins associated with bioenergetics and biosynthetic pathways, as well as that of some virulence factors, thus generating parasites that are more capable of successfully colonizing tissues and organs and reaching higher parasitic loads, thus causing organ damage.

The genetic variability of *T. cruzi* has been described from a virulence perspective [8]. However, differences between high- and low-virulence strains might correspond to inter-isolate variation rather than to actual differences related to virulence. To avoid this issue, a comparative analysis of two genetically related *T. cruzi* cell lines, exhibiting different virulence phenotypes, can help elucidate the pathogenic mechanisms of the parasite. In this study, C8C3***hvir*** induced high parasitaemia in mice, whereas C8C3***lvir*** induced low or subpatent parasitaemia. Phenotypic analysis of both cell lines enabled the identification of important differences: trypomastigotes derived from the C8C3***hvir*** cell line displayed a higher growth rate, increased cysteine proteinase activity, and greater resistance to the complement system. To identify differences at the protein level, the proteomes of both cell lines were analysed using nLC-MS/MS. Thus, these cell lines may serve as effective models for future research programs investigating *T. cruzi* virulence. In short, a defined set of biochemical pathways distinguished the C8C3***hvir*** cell line from C8C3***lvir***, although further studies are warranted to elucidate their functions in *T. cruzi* virulence.

In conclusion, differences in the virulence of *T. cruzi* strains go beyond the mere expression of virulence factors, either in the parasite membrane or as excretory/secretory molecules, including molecules loaded on extracellular vesicles. The study findings suggest that the virulence of *T. cruzi* involves the reprogramming of metabolic and biosynthetic pathways to ensure that mechanisms to bolster energy requirements are initiated, differential proteins are expressed, and protection is enhanced against oxidative stress. The upregulation of proteins, which was not observed in the low-virulence cell line, provides insights into the virulence of *T. cruzi* which could be extrapolated to other parasitic organisms. A deeper exploration of these pathways through omics and molecular biology approaches, such as Crisp/Cas9, would facilitate the identification of new therapeutic targets and provide potential vaccine candidates for Chagas disease.

## Data Availability

The mass spectrometry proteomics data have been deposited to the ProteomeXchange Consortium via the PRIDE partner repository with the dataset identifier P×D030117. Username: reviewer_pxd030117@ebi.ac.uk and Password: KfQViJwo.
